# Deoxynivalenol-3-Glucoside Content Is Highly Associated with Deoxynivalenol Levels in Two-Row Barley Genotypes of Importance to Canadian Barley Breeding Programs

**DOI:** 10.3390/toxins11060319

**Published:** 2019-06-05

**Authors:** James R. Tucker, Ana Badea, Richard Blagden, Kerri Pleskach, Sheryl A. Tittlemier, W. G. Dilantha Fernando

**Affiliations:** 1 Agriculture and Agri-Food Canada, Brandon Research and Development Centre, 2701 Grand Valley Road, P.O. Box 1000A, R.R. 3, Brandon, MB R7A 5Y3, Canada; ana.badea@canada.ca; 2 Department of Plant Science, 66 Dafoe Road, University of Manitoba, Winnipeg, MB R3T 2N2, Canada; Dilantha.Fernando@umanitoba.ca; 3 Grain Research Laboratory, Canadian Grain Commission, 303 Main St., Winnipeg, MB R3C 3G8, Canada; richard.blagden@grainscanada.gc.ca (R.B.); kerri.pleskach@grainscanada.gc.ca (K.P.); sheryl.tittlemier@grainscanada.gc.ca (S.A.T.)

**Keywords:** *Hordeum vulgare*, barley, Fusarium head blight, *Fusarium graminearum*, trichothecene, deoxynivalenol, deoxynivalenol-3-glucoside, DON, FHB

## Abstract

Barley (*Hordeum vulgare* L.) is a multipurpose crop that can be harvested as grain or cut prior to maturity for use as forage. Fusarium head blight (FHB) is a devastating disease of barley that reduces quality of grain. FHB can also result in the accumulation of mycotoxins such as deoxynivalenol (DON). Breeding FHB resistant varieties has been a long-term goal of many barley-producing countries, including Canada. While the genetic basis of DON detoxification via production of less-phytotoxic conjugates such as DON-3-glucoside (DON3G) is well documented in barley, little information exists in reference to varietal response. Over two years, 16 spring, two-row barley genotypes, of importance to western Canadian barley breeding programs, were grown as short-rows and inoculated following spike emergence with a *Fusarium graminearum* conidia suspension. Half of the plots were harvested at soft dough stage and then dissected into rachis and grain components, whereas the remainder was harvested at maturity. Multiple *Fusarium*-mycotoxins were assayed using liquid chromatography-mass spectrometry. Mycotoxin content was elevated at the earlier harvest point, especially in the rachis tissue. DON3G constituted a significant percentage (26%) of total trichothecene content and thus its co-occurrence with DON should be considered by barley industries. DON3G was highly correlated with DON and 3-acetyl-deoxynivalenol (3ADON). The ratio of D3G/DON exhibited consistency across genotypes, however more-resistant genotypes were characterized by a higher ratio at the soft-dough stage followed by a decrease at maturity. Plant breeding practices that use DON content as a biomarker for resistance would likely result in the development of barley cultivars with lower total DON-like compounds.

## 1. Introduction

Fusarium head blight (FHB) is a devastating disease of barley (*Hordeum vulgare* L.) and wheat (*Triticum aestivum* L.) that has led to immense economic losses [1]. In the USA, Canada, China, and southern and eastern Europe, this disease is primarily incited by *Fusarium graminearum* Schwabe (teleomorph *Gibberella zeae* (Schweinitz) Petch) [2], which produces a range of phytotoxic, secondary metabolites. *F. graminearum* is capable of producing multiple type B trichothecene mycotoxins, which are characterized by a keto (carbonyl) function at the C-8 position of the trichothecene molecule, eg. deoxynivalenol (DON), 3 and 15-acetyl deoxynivalenol (3ADON, 15ADON), nivalenol (NIV) [3]. Trichothecenes inhibit protein and ribonucleic acid synthesis, impact cellular functions of division, membranes and mitochondrial activity [4] and have potential to cause toxicoses in humans and animals [5]. *F. graminearum* also synthesizes zearalenone (ZEA) an immunotoxic compound that mimics estrogen by binding to mammalian receptors [6]. Trichothecenes such as DON are particularly damaging to agricultural production systems, due to their global distribution and the high frequency at which they occur [7]. Due to fact that DON is thermally stable and water soluble, it can readily be transmitted from malt to beer [8,9]. DON is highly regulated by the brewing and malting industry due to public safety concerns, where exceeding limits as low as 0.5 mg kg^−1^ may result in rejected sale [1]. Malting barley is considered a value-added crop with price premiums, where producers employ crop management strategies to minimize risk of quality loss due to the presence of DON. Breeding genetically resistant varieties remains the most economical and environmentally-friendly means to mitigate mycotoxin occurrence in human food and animal feed.

*F. graminearum* is considered a hemi-biotrophic pathogen. Following infection, the pathogen lives biotrophically within the host for a short period growing inter-cellularly and asymptomatically, then shortly thereafter switches to necrotrophy where it derives nutrients from dead cells [10,11]. The pathogen employs specialized infection structures such as compound appressoria and foot structures to penetrate the host tissues which are associated with DON production, regardless DON is not essential for the infection process [12]. Significant quantities of DON are not observed in barley until a few days post-infection, where it accompanies a transition to the necrotrophic phase [13,14]. Host plants have evolved layers of resistance mechanisms, which have been characterized as resistance categories for breeding purposes [15]. Resistance may involve the inhibition of initial infection (Type I); or restriction of subsequent spread (Type II) during the prolific phase, where a striking contrast exists for FHB epidemiology between barley and wheat [16]. Barley generally possesses superior Type II resistance [17]. In contrast to wheat, fungal spread is highly restricted at the rachis node and defense is not enhanced for mutants with a disrupted trichodiene synthase gene (*TRI5*) and inability to produce DON [18,19,20]. While DON does not contribute to fungal spread in barley, disrupted *tri5*-mutant strains produce lower disease severity, fungal biomass and floret necrosis/bleaching compared to the wild-type establishing the role of DON as a virulence factor [21]. Barley displays an extended infection period following anthesis, where later infections may be symptomless while still accumulating mycotoxin [22,23]. The sum of individual kernels from infections at different developmental stages collectively contribute to overall mycotoxin levels, both through mycotoxin content and grain biomass.

Following pathogen infection, plant defense may involve Type V resistance mechanisms (resistance to toxins) which may function to: a) modify mycotoxins via conjugation with other molecules for consequential detoxification and; b) limit mycotoxin production by the fungus [24]. Mycotoxins conjugated through specific reactions of host enzymes result in compounds with diminished phytotoxicity. Chemical modification of toxin structure may result in reduced toxicity by directly impeding their mode of action or increasing their mobility and transfer for storage in organs such as vacuoles and/ or binding to cellular membranes [25]. Once conjugated, modified mycotoxins may not be detected by routine analysis of food or feed products. Deoxynivalenol-3-glucoside (DON3G), a common conjugated form of DON found in barley grains, is readily cleaved in the intestines such that the same toxic effects are expected [26].

The genetic basis of DON detoxification mechanism has been well studied in barley, where a uridine diphosphate glycosyltransferase (UGT) barley gene *HvUGT13248* (MLOC_65675) has been functionally characterized through transgenic approaches in yeast [27] and *Arabidopsis thaliana* [28] model systems. Resistant wheat varieties that carry *Fhb1* QTL show greater ability for conversion of DON to DON3G in resistant genotypes [29,30]. However, in wheat lines with an elevated detoxification rate, levels of DON and DON3G together are generally lowered compared with susceptible varieties [31,32,33]. To date, minimal information is available regarding relative occurrence patterns of DON and its conjugated form DON3G and their interaction within barley in relation to differential resistance. To analyze DON, its conjugated form DON3G, and the acetylated forms of 3ADON and 15ADON, the current study utilized liquid chromatography-tandem mass spectrometry (LC-MS/MS). This is a method with high separation capacity of analytes paired with mass analysis which offers utility of multiplex analysis within an individual sample [34]. With increased understanding of the mechanisms that underlie resistance to DON accumulation, barley breeders can make informed decisions during development of FHB resistant varieties with the result of lowered toxin in the field. This study was conducted to evaluate the effect of genotypic differences in barley on mycotoxin production, with specific interest in DON3G and its relative occurrence to DON.

## 2. Results

This study evaluated a set of barley varieties that represent cultivars, elite breeding lines and FHB resistance sources relevant to the western Canadian barley breeding activities for developing barley cultivars with improved resistance to FHB (Table 1).

### 2.1. Heading, Incidence and Severity

The number of days to heading differed significantly amongst genotypes (F-value = 74.0, *p* ≤ 0.0001), while no significant interaction was observed between genotypes with year (F-value 1.62, *p* = 0.142). Genotypes demonstrated a 10 day range of average heading dates, where exotic breeding line HDE84194-622-1 headed at 45.0 ± 0.4 days in contrast with 55.3 ± 0.2 days for Canadian forage cultivar CDC Austenson. Control and inoculated treatment groups did not differ within any year (2016: *t* = 0.14; *p* = 0.889; 2017: *t* = 0.52; *p* = 0.600).

FHB was present at low levels in the control group each year, equivalent to background infection. A contrasting FHB epidemic was incited in the treatment group by the conidial spray applications. Difference of magnitude was observed between control and inoculated treatments (*p* ≤ 0.0001), for incidence (3.1% control vs. 33.5% treatment) and severity (1.8% control vs. 17.8% treatment). While FHB symptoms of control and Fusarium treatment groups differed by an order of magnitude, average incidence and severity of barley genotypes demonstrated positive association between the groups (*R*^2^ = 0.74; 0.61 respectively).

**Figure 1:**
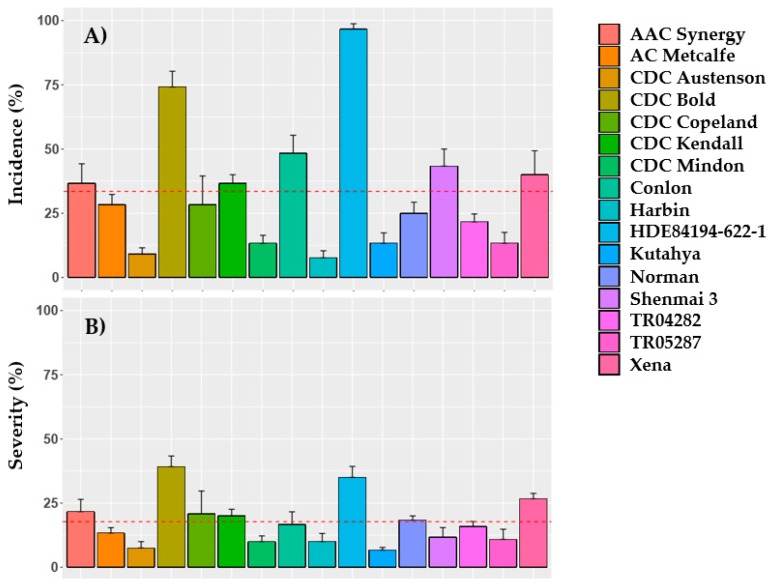
Fusarium head blight symptoms of 16 inoculated barley genotypes over 2016–2017 at Brandon, MB. (**A**) Percent incidence (**B**) Percent severity. Error bars represent standard error. Red dashed line represents overall mean.

FHB symptoms showed differentiation amongst barley genotypes with wide ranges in average percent incidence and severity, with less variation observed in the latter (Figure 1). Significant differences were observed amongst genotypes for incidence (F = 85.63, *p* ≤ 0.001) and severity (F = 27.08, *p* ≤ 0.001). A significant interaction was observed between genotype and year for incidence (F = 7.94, *p* ≤ 0.001) and severity (F = 6.11, *p* ≤ 0.001). A significant effect of year was seen for severity (F = 15.76, *p* = 0.017) but not for incidence (F = 4.23, *p* = 0.109). Highest incidence occurred in HDE84194-622-1, whereas semi-dwarf, Canadian variety CDC Bold displayed highest severity. Lowest incidence was observed for Chinese accession Harbin and lowest severity occurred in Kutahya, a cultivar from the Netherlands. Earlier heading genotypes such as Conlon, Shenmai 3 and HDE84194-622-1 have typically demonstrated higher resistance in past trials when inoculated using surface-applied, Fusarium-infected corn kernels [35].

### 2.2. Mycotoxins

Significant increases in mycotoxins were observed in the inoculated treatment group. Mycotoxins demonstrated a wide range of magnitude and frequency of occurrence (Supplementary Table S1 I-II). ZEA was found consistently but at very low levels, where a trend was observed for higher content at maturity (0.007 vs. 0.024 mg kg^−1^, *p* = 0.27). NIV was detected at low levels overall, but with higher concentration at early vs. mature stage (0.8 vs. 0.3 mg kg^−1^, *p* < 0.00001) and in rachis vs. grain tissues (2.1 vs. 0.8 mg kg^−1^, *p* < 0.0001). Culmorin (CUL) was only evaluated in 2017, where it showed low content in all tissues and stages (<1 mg kg^−1^ on average). 15ADON was only detected in the 2016 samples and at very low levels (<0.1 mg kg^−1^). Acetylated forms of DON ie. effectively 3ADON, were found at low levels representing approximately 4% of total DON for both control and inoculated treatments. In a few samples, 3ADON occupied a relatively larger proportion of total where it was found to represent up to 15% of DON-like compounds. 3ADON showed higher content at soft-dough (*p* < 0.0001) and in rachis vs. grain tissue (*p* < 0.01). As seen in general in the commercial samples, the majority of non-DON Fusarium-related mycotoxins were found at levels of insignificance even under epidemic conditions.

Of all the Fusarium-associated mycotoxins evaluated, DON and its conjugated form DON3G were detected commonly and at highest concentration levels, particularly for DON. Effect of genotype was significant for DON and DON3G in all tissues (Table 2A–C). DON and DON3G genotype means demonstrated moderate correlation between control and Fusarium treatment groups (*R*^2^ = 0.57 and 0.41 respectively). For inoculated materials, highest concentrations were observed at the earlier developmental stage and within rachis tissue (Table 3A–C). DON was detected above the limit of quantification (0.03 mg kg^−1^) for every inoculated sample, and in over 93% of the ripe grains of control treatment. Of the naturally infected control samples, 15% (2016) and 48% (2017) would have failed the industry malting acceptance standard of ≤0.5 mg kg^−1^ [1].

### 2.3. Relationships between Characters

**Figure 2:**
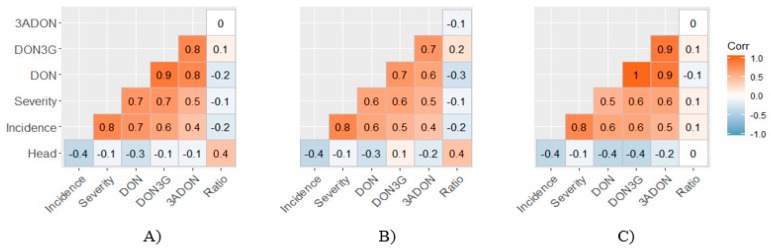
Pearson correlation coefficients for visual symptoms and mycotoxin content (mg kg^−1^), where Head = Days to 75% of spikes headed; Incidence = Percentage of infected spikes per plot; Severity = Percentage of infected grains per infected spike; DON = deoxynivalenol; DON3G = deoxynivalenol-3-glucoside; 3ADON = 3-acetyl-deoxynivalenol; Ratio = DON3G/DON. (**A**) grain tissue harvested at soft-dough stage. (**B**) rachis tissue harvested at soft-dough stage. (**C**) grain tissue harvested at maturity.

**Figure 3:**
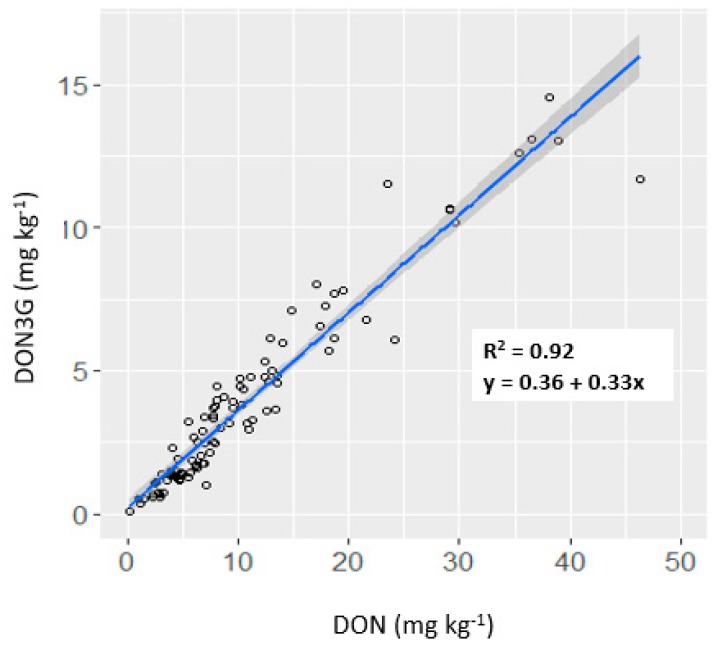
Linear relationship of deoxynivalenol-3-glucoside (DON3G) with deoxynivalenol (DON) content in matured grains. Shaded area represents 95% confidence interval for the fitted line.

Percent incidence and severity demonstrated a moderately-high positive association (Figure 2A–C). The number of days to heading was negatively associated with incidence and DON content. Both incidence and severity were moderately associated with DON content, where the strongest relationship was observed between visual symptoms and DON content in grains at the soft-dough stage. Incidence and severity demonstrated a moderate association with 3ADON. Visual symptoms were not associated with NIV and ZEA. DON3G demonstrated a very high affiliation with DON, which was highest in grains vs. rachis tissues (*R*^2^ = 0.92, Figure 3).

**Figure 4:**
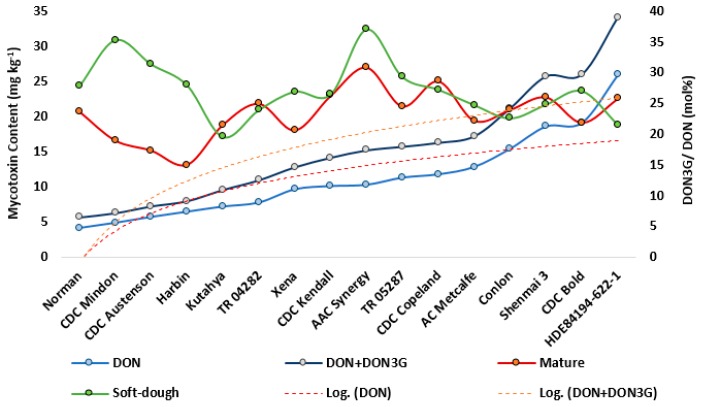
Left axis: Deoxynivalenol (DON) concentration (light blue line) and total DON + deoxynivalenol-3-glucoside (DON3G) concentration (black line) in grain; Corresponding logarithmic fit lines (broken lines). Right Axis: Ratio of DON3G to DON at soft-dough (green line) or mature stage (red line) for 16 barley genotypes organized in ascending order for mean DON content at maturity over 2016–2017.

Host resistance status, measured by FHB disease symptoms or DON content, did not appear to be strongly associated with the ratio of DON3G to DON (Figure 2A–C). This ratio proved consistent between soft-dough (27 mol%) and maturity harvest dates (23 mol%) (Figure 4). While the genotypes in this study displayed a wide range of resistance, the ratio generally displayed subtle differences over genotypes. However, some of the lowest-DON-producing genotypes such as Norman, CDC Mindon, CDC Austenson and Harbin demonstrated an above average ratio at soft-dough, but a below average ratio at maturity (Figure 4). At soft-dough harvest AAC Synergy and CDC Mindon displayed the highest DON3G/ DON ratio (37 mol% and 35 mol% respectively), whereas Kutahya and HDE84194-622-1 showed the lowest ratio (20 mol% and 22 mol% respectively). At maturity AAC Synergy and CDC Copeland displayed the highest DON3G/ DON ratio (31 mol% and 29 mol%), while Harbin and CDC Austenson showed the lowest ratio (15 mol% and 17 mol%). DON and DON3G were both found at elevated levels in the rachis tissue, where they were more equally represented (DON3G/ DON ratio = 71 mol%). Unlike in grain tissues, greater differentiation was seen over varieties where a negative relationship was observed for DON content at maturity and the ratio of DON3G/ DON (*R*^2^ = −0.37). HDE84194-622-1 and Shenmai 3 showed the lowest ratio (29 mol% and 39 mol% respectively), whereas Harbin and CDC Austenson displayed a very high ratio of (111 mol% and 95 mol%, respectively).

## 3. Discussion

A series of FHB epidemics of immense economic proportions occurred in the Red River basin of North America during the mid-1990s [36]. In Canada, FHB quickly arose from an insignificant concern, to the most economically damaging disease of barley in only a period of a few years [37]. From Manitoba, *F. graminearum* has spread into neighboring provinces of Saskatchewan and Alberta, which are the major regions of barley-production in Canada. Moderately-resistant germplasm has been identified in FHB nurseries under Canadian environmental conditions and developing FHB resistance with low DON accumulation has been a long-term goal maintained by the Canadian barley breeding programs [38]. Use of FHB resistant varieties that accumulate less DON in combination with proper crop management practices may increase the probability of barley being selected and sold with malting premium. This study investigated host response and mycotoxin accumulation of 16 two-row barley genotypes relevant to FHB resistance breeding activities for western Canada.

In Canada, 3ADON-producers of *F. graminearum* have rapidly displaced the traditional 15ADON population, where an east-to-west geographical gradient has been observed [39]. Several studies of wheat have shown that 3ADON chemotypes produce higher DON during pathogenesis [40,41,42]. A field-based barley study at the same location found higher DON content associated with the proportion of 3ADON-producers as measured by DNA markers [43]. Under the current study chemotypes were applied in equal proportion, however 3ADON was by far the dominant acetylated-form detected. DON content demonstrated a strong association with 3ADON under the study conditions. While many other virulence factors may be of equal importance, the competitiveness of 3ADON-producing strains may be associated with elevated DON production.

The relationship between infection and mycotoxin formation has not been extensively studied in barley [8]. The developing barley caryopsis is protected by the thick-walled epidermis of the palea and lemma, but the pathogen evades this barrier by entry via the crevice between these structures or apical mouth before fusion occurs approximately 8 days post-emergence of the spike [44,45]. Grains harvested at the soft-dough stage were observed to possess 22% higher DON content than those harvested at maturity. This is consistent with previous results for barley, where it has been demonstrated that DON content peaks during seed development, followed by detoxification at maturity [46]. Rachis tissue had the highest concentration of mycotoxins, 33% more DON than grains from same spikes. In wheat, elevated DON concentration has also been documented in rachis tissues [47]. In the current study, the ratio of DON3G to DON was much higher in the rachis (71 mol%) vs. grains tissue (27 mol%), indicating heightened resistance response in this tissue, which is in agreement with the strong Type II resistance seen in barley. While resistant to spread, barley is susceptible for an extended period following spike emergence, where cleistogamous (closed-flowered) genotypes accumulated more mycotoxins when inoculated 10 or 20 days after anthesis [22]. Recently, trichomes of palea and lemma have been highlighted as structural targets for susceptibility in later-stage infections [48]. Trichomes of rachis tissues have been previously hypothesized as an invasion point that could be used to overcome the strong Type II resistance of barley through external growth of mycelia over the epidermis [18]. Total-DON forms were elevated in rachis tissue both by higher DON levels and increased proportion of DON3G, however limitations of tissue biomass and impact of microbial activity in silage should be considered when determining risk of toxicosis when feeding animals forages produced using whole-plant tissues. Elevated DON and DON3G levels observed within the rachis may be the result of translocation from infected kernels or direct colonization. Regardless, this rachis tissue is highlighted for further study of host response in barley.

Transcriptomic analysis experiments of six-row barley which used contrasts of *tri5* mutants vs. wild type [21] or exogenous application of DON solution vs. mock [49] have identified a range of mycotoxin-specific genetic defense in barley. Induced genes were generally up-regulated during trichothecene accumulation, which included functions such as: transport, programed cell death, ubiquitination, transcription factors and cytochrome P450s and UGT genes. Wheat plants transformed with a barley UGT gene (*HvUGT13248*), exhibited elevated FHB resistance accompanied by reduced DON content through hastening elevation of DON3G/DON ratio [50]. Barley demonstrates a strong ability to induce an enzymatic system to convert DON to DON3G. An RNA-Sequencing analysis of near-isogenic lines (NILs) of Chevron-derived resistant alleles confirmed that *F. graminearum* infection-response in barley may involve both constitutive and inducible defense mechanisms involving earlier induction of genes such as *HvUGT13248* [51]. DON is known to interfere with ribosomal function and thereby production of defensive pathogen-related proteins, such that earlier onset of DON-related resistance may allow for expression of other resistance mechanisms. Fusarium resistance response is complex where defense compounds, typically associated with restricting fungal spread such as reactive oxygen species (ROS) such as H_2_O_2_, may ultimately be antagonistic due to positive regulation of trichothecene production [52]. For hemi-biotrophic organisms, functionality of defense response may depend on trophic status of the fungus and if it has shifted to necrotrophy for nutrient acquisition from dead tissue. Resistant plants may not only need resistance genes, but also the ability to regulate the defense response to the pathogen’s condition.

In matured grains, DON3G on average constituted one quarter (26%) of total-DON-like compounds (DON3G + 3ADON + DON), where a similar ratio was also observed in naturally infected controls. DON3G is known to be an important DON-form and has been detected in commercial samples from upper mid-western USA, however its relationship with DON has been inconsistent and subject to annual conditions [53]. The relationship between DON3G and DON has not been studied in barley within the context of varietal resistance. In this study, DON was found to be a robust predictor for total content of DON-like compounds and thus an adequate bio-indicator of quality for grain commerce while considering that it only constitutes a portion of total concentration. A very strong relationship was seen in the ratio of DON3G to DON, particularly within grain tissue. While both DON and DON3G content varied by resistance level of barley genotypes, their relative amounts appeared generally consistent. Genotypes with highest level of resistance were characterized by an elevated ratio of DON3G to DON at the soft-dough stage, followed by reduced representation of DON3G at maturity, implying that timing on expression of resistance response may be important. DON content used as a biomarker of resistance for selection, would likely facilitate lowering total-toxicity. DON3G constituted a significant portion of total concentration of DON-like compounds of plant tissues and this should be taken into consideration by barley industries.

## 4. Materials and Methods

### 4.1. Inoculum Preparation

An equal mixture of 3-ADON-producing (M2-06-01, Q-06-11, S3BS-06-01, WRS-2065 and WRS-2067) and 15-ADON-producing *F. graminearum* isolates (S3AN-06-01, NB-06-18, A1-06-01, WRS-1915 and WRS-1918) from multiple provinces across Canada were used to initiate inoculum. Mycelium-infected filter paper discs were placed on 10 cm plastic petri plates of potato dextrose agar (PDA). Petri plates were placed in a growth chamber for 10 days at 20 °C; 16 h D: 8 h L where they were grown to full colonization of the PDA media. Fresh tomatoes were cut into 1 cm^3^ cubes and 100 g L^−1^ were soaked in distilled water for 4 h. Solids were strained out through cheesecloth, and 15 g L^−1^ NaCl was added to the suspension. The suspension was divided into 500 mL aliquots in 1 L Erlenmeyer flasks, autoclaved for 20 min, and then allowed to cool. In a biosafety cabinet, individual-isolate PDA plates were cut into 1 cm^3^ cubes and placed into flasks. Flasks were placed on an orbital shaker for 2 weeks under natural light cycle. Following, conidia were counted on a hemocytometer under 100X microscopy, then diluted to 5 × 10^4^ spores m L^−1^. Tween 20 surfactant was added to the suspension at 0.04% (*v*/*v*). Standardized spore suspensions were mixed together in equal proportions for either chemotype set.

### 4.2. Field Experiment

A total of 16 two-row, spring barley varieties were selected for their differential patterns of resistance to FHB and DON accumulation, as previously assessed over multiple years in a FHB nursery at Brandon, MB. This set included ten cultivars adapted to western Canada (AAC Synergy, AC Metcalfe, CDC Austenson, CDC Bold, CDC Copeland, CDC Kendall, CDC Mindon, Conlon, Norman and Xena), two elite resistant Canadian breeder lines (TR04282 and TR05287) and four exotic barleys (Harbin, HDE84194-622-1, Kutahya and Shenmai 3). All varieties were grown together at the Agriculture and Agri-Food Canada, Brandon Research and Development Centre in 2016 (49°51′59.5″ N, 99°58′46.6″ W) and 2017 (49°51′55.5″ N, 99°58′52.9″ W). Research plots consisted of 0.9 m single rows with 0.3 m spacing. Plots were sown at a density of 40 seeds per row. The experimental design was a split-split plot arrangement randomized as a randomized complete block design. Whole plot treatments either consisted of inoculated plots of *F. graminearum* conidia suspensions or a mock spray of water and Tween 20. Sub-plots were barley genotypes, where the entire experiment was replicated 3 times. The entire experiment was surrounded by a double guard-row of tall, forage barley CDC Cowboy.

A polyvinyl chloride irrigation system was employed, consisting of a 5 cm main tube and 2 equi-spaced risers (10 cm) positioned above canopy. Fine water droplets were applied on a 7 m radius via NAAN 501-U 2.2 mm nozzle sprinkler heads. Moisture was applied prior to inoculations until spikes were saturated. Heading notes (75% of the row, spikes emerged) were taken on each plot. Following heading, plots were inoculated by 40 mL of conidia suspension or water as applied by spray wand from a stainless-steel canister pressured to 250 kPa. A second application was applied to each plot, three days following the first application.

Three weeks post infection, FHB incidence (% of spikes infected) and FHB severity (% of infected grains per infected per spike) were recorded, based on observations of 10 spikes. At soft-dough stage (Z85; Zadoks scale for growth stages of cereals [54]) half of the row was cut with a sickle and placed in a mesh harvest bag and held in a high capacity drier at 37 °C for three days to arrest fungal development. The remaining plot was allowed to ripen in situ and subsequently harvested at maturity (Z92) by a small-plot combine under a minimal wind speed setting.

### 4.3. Mycotoxin Analysis

#### 4.3.1. Sample Preparation

The spikes cut at soft-dough stage were further dissected into rachis and grain portions. Harvested grains from both developmental stages were sub-sampled by scooping 20 g, and ground to flour using a Perten 3610 laboratory mill. Likewise, rachis portions (1 g) were ground.

#### 4.3.2. Extraction and Assay

Samples were analyzed for mycotoxins following the method reported by Tittlemier et al. [55]. Grain samples were extracted using a 1:4 (*m*/*v*) ratio of extraction solution consisting of acetonitrile/Milli-Q water/acetic acid (74/25/1). Rachis samples were extracted using 1:8 (*m*/*v*) ratio of extraction solution. Samples were placed on a flatbed shaker for 20 min on high. After samples settled, the extract was transferred to a centrifuge tube and centrifuged at 20 °C, 2534× *g* for 10 min. A total of 62.5 uL of extract was diluted to a final volume of 1000 uL with 5 mM ammonium Acetate in water containing 0.5% acetic acid. Sample extracts were fortified with ^13^C-labeled internal standards to mitigate matrix effects during analysis. Mycotoxins were separated using reverse phase liquid chromatography and analyzed using tandem mass spectrometry in the electrospray ionization mode. Two transitions were monitored for each analyte. Analytes were considered to be identified and were quantified in samples when their retention time was within 0.1 min of the mean retention time in external standards used to construct the calibration curve, the ratio of qualifier to quantitation transition was within ±30% of the mean ratio in external standards used to construct the calibration curve, and the peak area signal-to-noise ratio was at least 10:1. A panel of fourteen mycotoxins (6 produced by *F. graminearum* in Canada) were quantified within grain and rachis tissues (Table S2–This table also provides the limit of quantification (LOQ)). Mycotoxins were quantified using a calibration curve constructed from 7 external standards. Peak areas from quantitation transitions were normalized to the peak area of ^13^C-labeled internal standard during data analysis in order to mitigate matrix effects on quantitation prior to interpolation of analyte concentration from the calibration curve. Specifically, ^13^C-labeled internal standards were available and used for DON and DON3G.

A number of quality control samples were processed and analyzed aside the study samples throughout the study to ensure good method performance throughout the multi-year study. Matrix blanks were used to monitor for contamination. Portions of matrix blanks were also fortified prior to extraction with all mycotoxin analytes, and were used with commercially-available and in-house reference materials to monitor the accuracy and precision of the method.

#### 4.3.3. Quality Control during Analysis

Calibration curves (*R*^2^ > 0.99) were used to calculate the concentration of analyte. Analyte concentrations were recovery corrected using the percent recovery of analyte from a matrix-matched fortified blank, which was run with each batch of samples. Matrix-matched blanks, matrix-matched fortified blanks (spiked with a solution of standards) and certified reference materials were extracted and analyzed to monitor method performance throughout this study.

### 4.4. Statistical Analysis

A generalized linear mixed model (GLMM), was used to analyze the data over years. PROC GLIMMIX in SAS (version 9.4; SAS Institute, Cary, NC, USA) was used to test for significant differences in genotypes across years for FHB incidence and FHB severity and mycotoxin content. Replicate was nested in year as a random factor and modeled using a Poisson distribution and log link function. Mycotoxin content (DON, DON3G, 3ADON, DON3G/DON) were modeled using a log normal distribution and identity link function. Tukey-Kramer honestly significant difference was used to test for all pairwise comparisons.

Data were converted from mass to molar concentrations through division by molar mass of DON3G and DON (458.5 g mol^−1^ and 298.3 g mol^−1^ respectively) to facilitate comparison of DON3G/ DON ratios amongst genotypes. This allowed DON3G/ DON ratios to be reported on a molar basis, as opposed to a mass basis. Ratios were calculated as DON3G mol/ DON mol × 100.

‘cor’ function in R computing environment (R core team) was used to generate Pearson correlations between days to heading, FHB incidence, FHB severity, DON, DON3G, 3ADON, DON3G/DON for 16 genotypes. Packages ‘ggcorrplot’ and ‘ggplot2’ were used to produce graphics of the correlation matrix heatmap and scatterplot.

## Figures and Tables

**Table 1:** Pedigree and origin of 16 two-row, hulled spring barley genotypes evaluated for reaction to Fusarium head blight in 2016 and 2017 at Brandon, Manitoba.

Name (Alias ^1^)	Pedigree	Origin ^2^	Type ^3^	Release ^4^
AAC Synergy (TR09208)	TR 02267/Newdale	CDN	MALT	2012
AC Metcalfe (TR232)	AC Oxbow/Manley	CDN	MALT	1997
CDC Austenson (TR06389)	TR358/94Ab12271	CDN	GP/F	2010
CDC Bold (SD422)	SB88403/Tyra	CDN	GP	1999
CDC Copeland (TR150)	WM861-5/TR118	CDN	MALT	1999
CDC Kendall (TR133)	Manley/SM85221	CDN	MALT	1999
CDC Mindon (TR04378)	TR339/TR251	CDN	GP	2008
Conlon (TR982)	Bowman*2/Birgitta dwarf//ND10232	USA	MALT ^5^	2004
Harbin		CHN	-	-
HDE84194-622-1		CHN	-	-
Kutahya		NLD	-	-
Norman (TR05915)	In vitro selection from CDC Kendall	CDN	MALT	2009
Shenmai 3	Gobernadora /Humai 10	CHN	-	-
TR04282	Harbin/TR253//TR253	CDN	MALT	-
TR05287	Svanhals/AC Metcalfe//TR253	CDN	MALT	-
Xena (TR975)	Stark/Baroness	USA	GP	2002

^1^ Coding under evaluation in Western Canadian Cooperative Barley Registration Test. ^2^ CDN = Canada; CHN = China; NLD = Netherlands; USA = United States of America. ^3^ MALT = Malting; GP = General Purpose; GP/F = General Purpose, Forage. ^4^ Year of rights granted by Canadian Food Inspection Agency. Conlon was registered in USA in 1996. ^5^ Accepted for malting quality in USA but not in the Canadian market.

**Table 2:** Analysis of variance (ANOVA) for deoxynivalenol (DON), deoxynivalenol-3-glucoside (DON3G), 3-acetyl-deoxynivalenol (3ADON) concentration and the ratio (DON3G/DON) for different sample types.

Source	DON		DON3G		3ADON		DON3G/DON	
	F	Pr > F	F	Pr > F	F	Pr > F	F	Pr > F
**(A) Grain–Soft-dough**								
Genotype	4.09	<0.0001	4.72	<0.0001	5.27	<0.0001	2.21	0.02
Year	2.96	0.16	5.58	0.08	16.92	0.01	6.74	0.01
Genotype x Year	2.17	0.02	2.51	0.01	9.15	<0.0001	1.11	0.37
**(B) Rachis–Soft-dough**								
Genotype	3.63	0.0002	2.43	0.01	2.12	0.03	4.72	<0.0001
Year	5.81	0.07	1.38	0.3	3.43	0.07	12.01	<0.001
Genotype x Year	1.5	0.13	2.1	0.02	5.24	< 0.0001	3.51	<0.001
**(C) Grain–Mature**								
Genotype	4.31	<0.0001	5.56	<0.0001	3.14	0.001	9.51	<0.0001
Year	5.36	0.08	10.07	0.03	16.39	0.02	23.04	<0.0001
Genotype x Year	3.91	<0.0001	2.53	0.01	4.32	<0.0001	3.09	<0.001

**Table 3:** Least-squares means of 16 barley genotypes for deoxynivalenol (DON), deoxynivalenol-3-glucoside (DON3G), 3-acetyl-deoxynivalenol (3ADON) concentration (mg kg^−1^) and the percent molar ratio (DON3G/DON) for different sample types over years 2016–2017.

Genotype	DON		DON3G		3ADON		DON3G/DON	
**(A) Grain–Soft-dough**								
AAC Synergy	19.5	abc	11.2	a	1.5	a	37%	a
AC Metcalfe	12.9	abc	4.9	ab	1.4	a	25%	ab
CDC Austenson	4.2	bc	2.1	ab	0.4	a	31%	ab
CDC Bold	33.2	ab	14.0	a	2.8	a	27%	ab
CDC Copeland	9.0	abc	3.3	ab	0.6	a	27%	ab
CDC Kendall	11.0	abc	4.4	ab	1.0	a	26%	ab
CDC Mindon	4.0	c	2.7	b	0.5	a	35%	ab
Conlon	17.3	abc	6.0	ab	0.9	a	23%	ab
Harbin	9.2	c	2.5	b	0.8	a	28%	ab
HDE84194-622-1	32.8	a	10.9	a	1.8	a	22%	ab
Kutahya	5.8	abc	1.8	b	0.5	a	20%	b
Norman	3.8	c	1.8	b	0.3	a	28%	ab
Shenmai 3	17.8	abc	5.7	ab	1.4	a	25%	ab
TR 04282	14.4	abc	6.0	ab	1.5	a	24%	ab
TR 05287	14.7	abc	6.4	ab	1.3	a	29%	ab
Xena	11.4	abc	4.4	ab	1.0	a	27%	ab
Overall Mean	13.8		5.5		1.1		27%	
**(B) Rachis–Soft-dough**								
AAC Synergy	34.0	ab	40.1	a	2.3	a	89%	abc
AC Metcalfe	17.7	abc	15.2	ab	1.8	a	73%	abc
CDC Austenson	6.1	bc	7.4	ab	0.8	a	95%	ab
CDC Bold	28.7	abc	37.7	a	2.5	a	88%	abc
CDC Copeland	13.0	abc	13.7	ab	0.8	a	69%	abc
CDC Kendall	15.8	abc	16.3	ab	1.3	a	99%	abc
CDC Mindon	8.6	abc	8.0	ab	1.0	a	75%	abc
Conlon	26.5	abc	10.1	ab	1.1	a	48%	cd
Harbin	12.8	c	11.8	ab	1.1	a	111%	a
HDE84194-622-1	48.8	a	22.9	ab	3.7	a	29%	d
Kutahya	5.6	bc	5.9	b	0.3	a	57%	bcd
Norman	5.1	c	5.6	ab	0.3	a	85%	abc
Shenmai 3	17.2	abc	9.2	ab	1.0	a	35%	cd
TR 04282	18.1	abc	16.5	ab	1.9	a	60%	abcd
TR 05287	20.3	abc	17.8	ab	1.7	a	65%	abc
Xena	16.6	abc	15.8	ab	1.5	a	61%	abc
Overall Mean	18.4		15.9		1.5		71%	
**(C) Grain–Mature**								
AAC Synergy	10.3	abc	4.9	abcd	0.6	ab	31%	a
AC Metcalfe	12.9	abc	4.4	abcd	0.7	ab	22%	bcd
CDC Austenson	5.7	bc	1.6	cde	0.3	b	17%	f
CDC Bold	19.2	ab	6.9	abc	1.2	a	22%	bcd
CDC Copeland	11.8	abc	4.5	abcde	0.8	ab	29%	ab
CDC Kendall	10.1	abc	4.1	abcde	0.6	ab	26%	abc
CDC Mindon	4.9	c	1.4	e	0.3	ab	19%	df
Conlon	15.5	ab	5.8	ab	0.6	ab	23%	abcd
Harbin	6.5	bc	1.5	d	0.3	ab	15%	f
HDE84194-622-1	26.0	a	8.2	a	1.1	a	26%	abcd
Kutahya	7.2	abc	2.4	abcde	0.3	ab	22%	bcd
Norman	4.1	c	1.6	bcde	0.2	b	24%	abcd
Shenmai 3	18.6	ab	7.1	a	0.7	ab	26%	abc
TR 04282	7.8	abc	3.2	abcde	0.6	ab	25%	abcd
TR 05287	9.9	abc	3.9	abcde	0.5	ab	25%	abcd
Xena	9.7	abc	3.2	abcde	0.5	ab	21%	cd
Overall Mean	11.3		4.0		0.6		23%	

Note: Letter following represents significance group from Tukey-Kramer Honest Significant Difference test), where shared letters indicate values that do not differ significantly. Means have been back-transformed to original scale.

## Supplementary Materials

The following are available online at https://www.mdpi.com/2072-6651/11/6/319/s1, Table S1-I: Descriptive statistics of mycotoxin concentrations (mg kg^−1^) of mock-inoculated (water) barley plots by developmental stage at harvest and tissue type in 2016 and 2017. Table S1-II: Descriptive statistics of mycotoxin concentrations (mg kg^−1^) of Fusarium-inoculated barley plots by developmental stage at harvest and tissue type in 2016 and 2017. Table S2: Panel of fourteen mycotoxins and their limit of quantification for samples evaluated in 2016 and 2017 for content in barley tissues.
