# Supplementary material for: Deoxynivalenol-3-Glucoside Content Is Highly Associated with Deoxynivalenol Levels in Two-Row Barley Genotypes of Importance to Canadian Barley Breeding Programs
Source: Toxins (Basel). 2019 Jun 5;11(6):319. doi: 10.3390/toxins11060319 (PMC6628427; doi:10.3390/toxins11060319)
Supplement: Supplementary file 1 [file toxins-11-00319-s001.pdf]

Supplementary Materials: Deoxynivalenol-3-Glucoside Content is Highly Associated with Deoxynivalenol Levels in Two-Row Barley Genotypes of Importance to Canadian Barley Breeding Programs

James R. Tucker *, Ana Badea, Richard Blagden, Kerri Pleskach, Sheryl A. Tittlemier and W.G. Dilantha Fernando

Table S1-I. Descriptive statistics of mycotoxin concentrations (mg kg^{-1}) of mock-inoculated (water) barley plots by developmental stage at harvest and tissue type in 2016 and 2017 (n = 48).

(A)	Grain-Soft-dough	2016				2017			
	**Mycotoxin** [1]	**mean**	**se**	**min**	**max**	**mean**	**se**	**min**	**max**
	HT2	0.00	0.00	0.00	0.00	0.00	0.00	0.00	0.00
	T2	0.00	0.00	0.00	0.00	0.00	0.00	0.00	0.00
	15ADON	0.00	0.00	0.00	0.12	0.00	0.00	0.00	0.00
	DON3G	0.09	0.02	0.00	0.53	0.25	0.05	0.00	1.72
	NIV	0.83	0.13	0.00	4.02	0.91	0.13	0.00	5.07
	DON	0.24	0.07	0.00	2.89	0.54	0.11	0.00	4.23
	ZEA	0.00	0.00	0.00	0.00	0.00	0.00	0.00	0.03
	3ADON	0.01	0.00	0.00	0.18	0.05	0.01	0.00	0.62
	CUL	-	-	-	-	0.06	0.01	0.00	0.46
**(B)**	**Rachis-Soft-dough**	**2016**				**2017**			
	**Mycotoxin**	**mean**	**se**	**min**	**max**	**mean**	**se**	**min**	**max**
	HT2	0.01	0.01	0.00	0.26	0.00	0.00	0.00	0.00
	T2	0.00	0.00	0.00	0.08	0.00	0.00	0.00	0.00
	15ADON	0.01	0.01	0.00	0.36	0.00	0.00	0.00	0.00
	DON3G	0.36	0.09	0.00	2.98	0.70	0.09	0.00	2.34
	NIV	2.54	0.44	0.00	15.81	0.95	0.24	0.00	7.26
	DON	0.35	0.09	0.00	3.10	0.63	0.20	0.00	9.37
	ZEA	0.00	0.00	0.00	0.00	0.00	0.00	0.00	0.00
	3ADON	0.02	0.01	0.00	0.32	0.05	0.02	0.00	0.42
	CUL	-	-	-	-	0.13	0.02	0.00	0.61
**(C)**	**Grain-Mature**	**2016**				**2017**			
	**Mycotoxin**	**mean**	**se**	**min**	**max**	**mean**	**se**	**min**	**max**
	HT2	0.00	0.00	0.00	0.06	0.00	0.00	0.00	0.00
	T2	0.00	0.00	0.00	0.00	0.00	0.00	0.00	0.00
	15ADON	0.00	0.00	0.00	0.00	0.00	0.00	0.00	0.00
	DON3G	0.10	0.02	0.00	0.44	0.19	0.03	0.00	0.92
	NIV	0.25	0.03	0.00	1.05	0.28	0.04	0.00	1.40
	DON	0.29	0.04	0.00	1.46	0.61	0.07	0.00	2.27
	ZEA	0.00	0.00	0.00	0.10	0.00	0.00	0.00	0.00
	3ADON	0.00	0.00	0.00	0.08	0.03	0.01	0.00	0.19
	CUL	-	-	-	-	0.07	0.01	0.01	0.30

[1] HT2 = HT2 toxin; T2 = T2 toxin; 15ADON = 15-acety-ldeoxynivalenol; DON3G = deoxynivalenol-3-glucoside; NIV = nivalenol; DON = deoxynivalenol; ZEA = zearalenone; 3ADON = 3-acetyl-deoxynivalenol; CUL = culmorin. se = standard error; min=minimum; max=maximum.

Table S1-II. Descriptive statistics of mycotoxin concentrations (mg kg^{-1}) of Fusarium-inoculated barley plots by developmental stage at harvest and tissue type in 2016 and 2017 (n = 48).

(A)	Grain-Soft-dough	2016				2017			
	**Mycotoxin** [1]	**mean**	**se**	**min**	**max**	**mean**	**se**	**min**	**max**
	HT2	0.00	0.00	0.00	0.00	0.01	0.01	0.00	0.35
	T2	0.00	0.00	0.00	0.00	0.01	0.01	0.00	0.31
	15ADON	0.09	0.02	0.00	0.83	0.00	0.00	0.00	0.00
	DON3G	6.91	0.83	0.10	22.77	4.12	0.63	0.07	22.38
	NIV	0.77	0.12	0.00	4.20	0.83	0.08	0.00	2.21
	DON	16.09	1.89	0.11	57.99	11.59	1.87	0.15	62.98
	ZEA	0.01	0.01	0.00	0.31	0.01	0.00	0.00	0.07
	3ADON	1.50	0.20	0.00	5.35	0.72	0.15	0.00	4.51
	CUL	-	-	-	-	0.89	0.17	0.02	5.59
**(B)**	**Rachis-Soft-dough**	**2016**				**2017**			
	**Mycotoxin**	**mean**	**se**	**min**	**max**	**mean**	**se**	**min**	**max**
	HT2	0.04	0.02	0.00	1.11	0.00	0.00	0.00	0.00
	T2	0.00	0.00	0.00	0.04	0.00	0.00	0.00	0.00
	15ADON	0.10	0.02	0.00	0.54	0.00	0.00	0.00	0.00
	DON3G	19.47	2.32	0.17	58.35	12.35	2.13	0.00	71.81
	NIV	3.30	0.59	0.00	17.22	0.92	0.17	0.00	4.27
	DON	23.92	2.93	0.11	101.92	12.93	2.24	0.33	63.45
	ZEA	0.02	0.01	0.00	0.26	0.01	0.01	0.00	0.18
	3ADON	1.89	0.23	0.00	5.38	1.02	0.27	0.00	10.19
	CUL	-	-	-	-	1.86	0.33	0.18	10.13
**(C)**	**Grain-Mature**	**2016**				**2017**			
	**Mycotoxin**	**mean**	**se**	**min**	**max**	**mean**	**se**	**min**	**max**
	HT2	0.00	0.00	0.00	0.00	0.00	0.00	0.00	0.11
	T2	0.00	0.00	0.00	0.00	0.00	0.00	0.00	0.00
	15ADON	0.01	0.01	0.00	0.18	0.00	0.00	0.00	0.00
	DON3G	4.59	0.43	1.17	13.10	3.49	0.55	0.34	15.51
	NIV	0.24	0.02	0.06	1.15	0.30	0.03	0.00	1.05
	DON	11.89	1.13	3.79	38.91	10.67	1.75	0.97	54.62
	ZEA	0.03	0.01	0.00	0.20	0.02	0.01	0.00	0.52
	3ADON	0.74	0.09	0.21	3.27	0.43	0.07	0.00	2.72
	CUL	-	-	-	-	0.64	0.10	0.06	2.74

[1] HT2 = HT2 toxin; T2 = T2 toxin; 15ADON = 15-acety-ldeoxynivalenol; DON3G = deoxynivalenol-3-glucoside; NIV = nivalenol; DON = deoxynivalenol; ZEA = zearalenone; 3ADON = 3-acetyl-deoxynivalenol; CUL = culmorin. se = standard error; min=minimum; max=maximum.

Table S2. Panel of fourteen mycotoxins and their limit of quantification for samples evaluated in 2016 and 2017 for content in barley tissues.

Abbreviation	Mycotoxin	LOQ [1]
DON	deoxynivalenol	0.03
DON-3G	deoxynivalenol-3-glucoside	0.05
15-ADON	15-acetyl deoxynivalenol	0.05
3-ADON	3-acetyl deoxynivalenol	0.05
ZEA	zearalenone	0.02
NIV	nivalenol	0.06
CUL [2]	culmorin	0.05
HT-2	HT-2 toxin	0.06
T-2	T-2 toxin	0.03
AFG1	aflatoxin G2	0.001
AFG2	aflatoxin G1	0.001
AFB1	aflatoxin B2	0.001
AFB2	aflatoxin B1	0.001
OTA	ochratoxin A	0.0006

[1] Limit of quantification (mg kg^{-1}); [2] Only evaluated in 2017.
